# A novel *ELF4* gene variant disrupts T and NK cell function in a patient with immune thrombocytopenia (ITP)

**DOI:** 10.1007/s00011-026-02270-1

**Published:** 2026-05-19

**Authors:** Perihan Kader Kendirli, Şerife Erdem, Ayşenur Paç Kısaarslan, Veysel Gök, Eda Kayhan, Alper Özcan, Muhammet Ensar Dogan, Christoph Klein, Ekrem Ünal, Ahmet Eken

**Affiliations:** 1 Department of Bioengineering, Institute of Engineering and Natural Sciences, Abdullah Gül University, Kayseri, Turkey; 2 Genome and Stem Cell Center (GENKOK), Erciyes University, Kayseri, Turkey; 3 Department of Immunology, Faculty of Medicine, Kırşehir Ahi Evran University, Kırşehir, Turkey; 4 Division of Pediatric Rheumatology, Faculty of Medicine, Erciyes University, Kayseri, Turkey; 5 Division of Pediatric Hematology and Oncology, Faculty of Medicine, Erciyes University, Kayseri, Turkey; 6 Division of Medical Genetics, Kayseri City Hospital, Kayseri, Turkey; 7 Department of Pediatrics, Dr. von Hauner Children’s Hospital, University Hospital, Ludwig Maximilian University of Munich, Munich, Germany; 8 German Center for Child and Adolescent Health (DZKJ), Munich Site, Munich, Germany; 9 Hasan Kalyoncu University, School of Health Sciences, Deparment of Nursing, 27410 Gaziantep, Turkey; 10 Erciyes University School of Medicine, Department of Medical Biology, 38030 Kayseri, Turkey; 11 Department of Immunology and Microbiology, University of Colorado Anschutz Medical Campus, Aurora, CO USA

**Keywords:** ELF4, ITP, Deficiency in ELF4, X-linked

## Abstract

**Objective and design:**

In this report, we identified a novel hemizygous *ELF4* variant (c.1822G > C; p.Gly608Arg) in an adolescent male with chronic immune thrombocytopenia (ITP) and performed functional immunologic characterization.

**Materials and methods:**

Peripheral blood mononuclear cells (PBMCs) of the patient and age-matched controls were characterized by flow cytometry with respect to T cell phenotype, activation, proliferation and NK cell cytotoxicity.

**Results:**

The p.Gly608Arg substitution affects a highly conserved residue in the C-terminal regulatory domain of *ELF4* and is predicted to be damaging. Immunophenotyping showed an expanded CD8^+^ T-cell compartment, an inverted CD4/CD8 ratio, reduced naïve T-cell populations, and accelerated acquisition of memory-like phenotypes upon activation. Both CD4^+^ and CD8^+^ T cells displayed increased proliferation following TCR stimulation, consistent with impaired *ELF4*-dependent regulation of effector T-cell expansion. NK cells exhibited reduced granzyme B and perforin expression and markedly diminished cytotoxicity against K562 targets, indicating defects in maturation and effector function.

**Conclusions:**

These findings suggest that the identified ELF4 variant is associated with combined T- and NK-cell dysfunction. This case expands the clinical spectrum of Deficiency in *ELF4*,* X-linked* and underscores the relevance of evaluating *ELF4* mutations in patients with unexplained cytopenias accompanied by dysregulated lymphocyte activation and impaired cytotoxic responses.

**Supplementary Information:**

The online version contains supplementary material available at 10.1007/s00011-026-02270-1.

## Introduction

A recently identified inborn error of immunity, Deficiency in *E74-like ETS family transcription factor 4* (*ELF4*), X-linked is typified by immune dysregulation, persistent mucosal autoinflammation, and susceptibility to infection and clinically *ELF4*-deficient cases are characterized by recurrent respiratory infections and autoinflammatory disease of the digestive tract, such as oral/ileocecal/anal ulcers [1–3]. *ELF4*, encoded by Xq26, integrates innate and adaptive programs by regulating natural killer (NK) cell development and function [4, 5] cell-cycle arrest in naïve CD8^+^ T cells, the development and maintenance of memory CD8^+^ T cells [6], type I interferon–responsive pathways [7], and T_H_17 cell differentiation [8]. Functional studies demonstrate that *ELF4* is a key regulator of immune homeostasis, as loss-of-function variants of the gene suppress the transcription of anti-inflammatory genes while increasing pro-inflammatory responses. Nevertheless, due to the limited number of confirmed cases [1–3], the phenotypic spectrum and cytotoxic effector consequences of *ELF4* dysfunction in humans remain poorly understood. In this context, we examined NK- and T-cell immunity in a patient harboring a hemizygous *ELF4* c.1822G > C (p.Gly608Arg) variant to clarify how cytotoxic effector programs and inflammatory control are perturbed by *ELF4* dysfunction.

## Case report

We report here a 14-year-old male with immune thrombocytopenic purpura and persistent thrombocytopenia, initially diagnosed at the age of 10 years. Despite treatment with intravenous immunoglobulin and corticosteroids, thrombocytopenia persisted. Subsequent therapies, including romiplostim, rituximab, and eltrombopag, resulted only in transient and insufficient responses without sustained remission, supporting a chronic refractory disease course. At the most recent follow-up, the patient remained thrombocytopenic while continuing eltrombopag treatment but had no active bleeding manifestations. The patient’s medical history revealed no recurrent or severe infections and no previous hospitalizations related to infectious complications. Notably, since early childhood, he had experienced recurrent and prolonged ulcerative lesions involving the lips, together with intermittent aphthous ulcers of the oral mucosa, suggesting mild mucosal inflammatory involvement. He had no history of chronic enteritis or inflammatory bowel symptoms. Additionally, there was no family history of primary immunodeficiency, autoimmune disease, or early unexplained deaths. Apart from immune thrombocytopenia and these recurrent mucosal lesions, no other clinical manifestations suggestive of overt immunodeficiency were identified until to the time of evaluation.

Complete blood count assessment revealed normal WBC 7980/µL, neutrophils 4280/µL, lymphocytes 2400/µL, eosinophils 520/µL (6.5%), hemoglobin 15 g/dL, and reduced platelets 5000/µL (reference range: 150000/µL − 400000/µL). Baseline immunoglobulin levels were as follows: IgG 1011 mg/dL, IgA 134 mg/dL, IgM 58 mg/dL, and IgE 63 IU/mL. Age-adjusted reference ranges for adolescents are: IgG 700–1600 mg/dL, IgA 70–400 mg/dL, IgM 70–250 mg/dL, and IgE 0–100 IU/mL. Accordingly, IgM levels were mildly reduced, while other immunoglobulin levels were within normal limits.

Lymphocyte subset analysis showed CD3^+^ 77.9% (2714/µL), CD4^+^ 33.4% (1148/µL), CD8^+^ 33.8% (1148/µL), NK cells 6.3% (219/µL), and CD19^+^ 11.6% (382/µL). Age-matched reference ranges are: CD3^+^ 60–85%, CD4^+^ 30–60%, CD8^+^ 15–40%, CD19^+^ 5–20%, and NK cells 5–20%. Based on these values, lymphocyte subset distribution was largely within normal limits; however, the CD4/CD8 ratio was at the lower end of the expected range.

Evaluation for common secondary causes of chronic thrombocytopenia did not reveal clinical evidence of systemic lupus erythematosus, connective tissue disease, or other secondary autoimmune conditions. Autoimmune screening showed negative ANA and anti-dsDNA, normal complement levels (C3 159 mg/dL, C4 24.4 mg/dL), RF 7.7 IU/mL, negative direct Coombs test, and negative antiphospholipid antibodies; ENA/myositis panel was largely unremarkable except for isolated anti-PM-Scl positivity and weak anti-SSA (Ro52) reactivity without clinical correlation. Given the combination of childhood-onset refractory ITP, mildly reduced IgM levels, and subtle abnormalities in lymphocyte subset distribution, an underlying inborn error of immunity was considered, and whole-exome sequencing (WES) was performed.

**Fig. 1:**
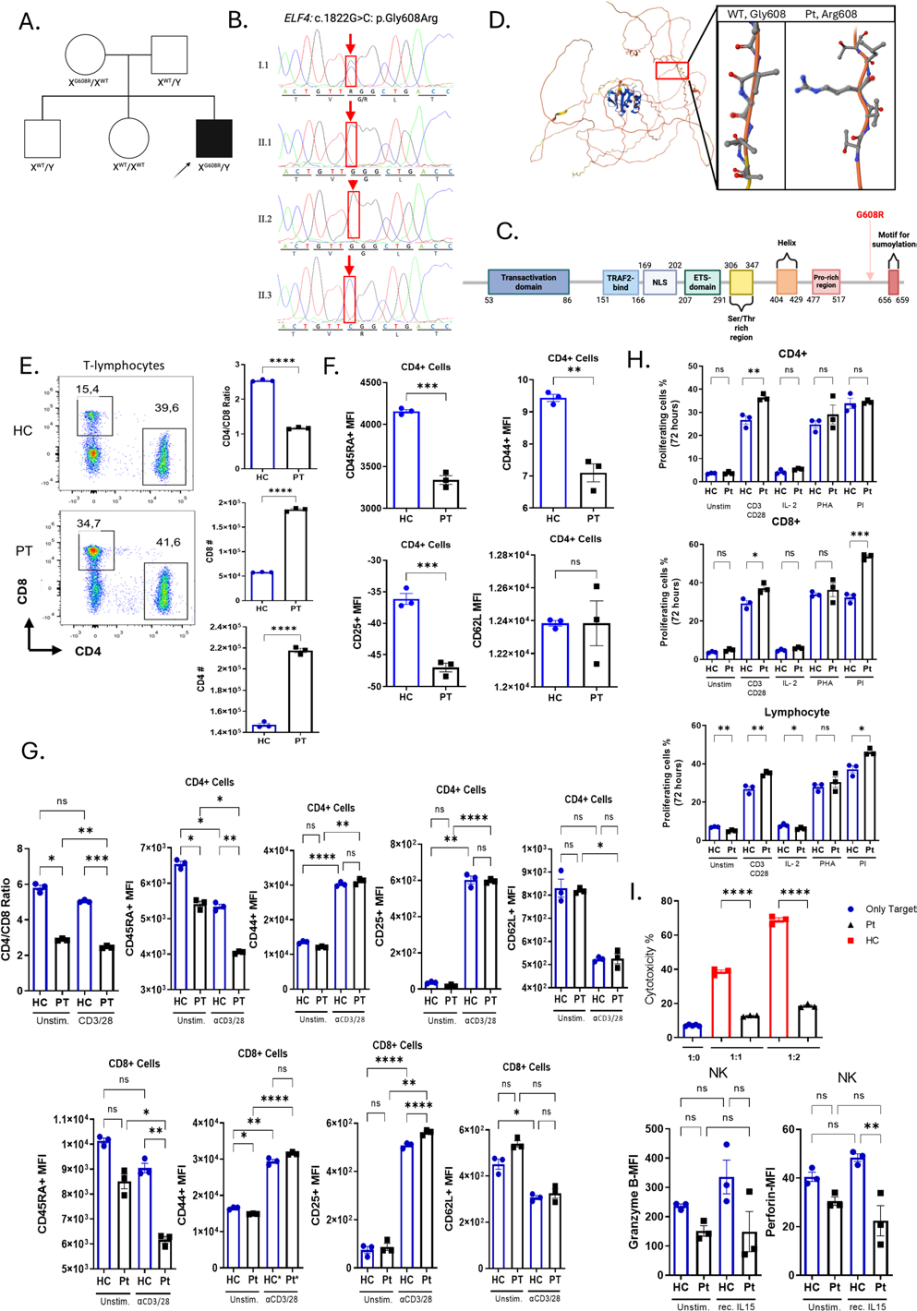
ELF4 p.Gly608Arg is a loss of function. A Pedigree of the family showing segregation of the p.G608R variant, with the patient represented in black and the healthy individuals in white. Genotypes are indicated below each individual. The proband is marked with an arrow. B Sanger sequencing chromatograms showing the c.1822G > C substitution in the carrier mother (heterozygous), unaffected siblings (WT), and proband (hemizygous). The arrow displays the nucleotide substitution. C Schematic of ELF4 protein structure, highlighting the location of p.Gly608Arg mutation within the C-terminal region. D Ribbon model of the human ELF4 protein created via AlphaFold web viewer (UniProt Q99607), with the p.G608R variant highlighted in red. E Representative flow cytometry dot plots demonstrate CD4^+^ and CD8^+^ T cell populations from the peripheral blood of a healthy control (top panel) and the patient (bottom panel), which were gated on CD3^+^ T lymphocytes. Quantified MFI plots of CD4+ (middle) and CD8 + T (bottom) cells from PBMCs and CD4/CD8 Ratio (upper). F Representative MFI plots illustrating CD45RA, CD44, CD25, and CD62L expression on CD4^+^ T cells from the PBMCs isolated from the patient and healthy control. G PBMCs from the patient and healthy control were cultured in triplicate and stimulated overnight with or without anti-CD3/CD28. The diminished CD4/CD8 cell ratio for the patient is depicted in the MFI plot (left panel). Quantifying mean fluorescence intensity (MFI) for CD45RA, CD44, CD25, and CD62L on CD4^+^ T and CD8^+^ T cells. HC (blue circles) and Pt (black squares) are shown. H PBMCs isolated from the patient and healthy control were labeled with CFSE and stimulated for 72 h in complete medium under distinct stimulatory conditions. Subsequently, cell proliferation was assessed by flow cytometry. Proliferation of CD4 + T cells (left panel), CD8 + T cells (middle panel), and lymphocytes (right panel) is represented in bar graphs. Statistical comparisons were performed between the PT and HC groups under each stimulation condition. I PBMCs from the patient, and HC were co-cultured with labeled K562 target cells at distinct effector-to-target (E: T) ratios as 1:0, 1:1, and 1:2. The death in the target cells stained with ANNEXIN V and 7-AAD was quantified (upper plot). MFI plots of Granzyme-B and Perforin expression in sorted NK cells from the patient and healthy control are shown. Samples from the healthy control and patient were run in triplicate. Indicate *P < 0.05, **P < 0.01, ***P < 0.001, ****P < 0.0001. The error bars indicate ± SEM. HC: Healthy Control, PT: Patient

WES revealed a novel hemizygous missense variant in the X-linked transcription factor *ELF4* gene (NM_001421.4: c.1822G > C; p.Gly608Arg). It was discovered that this alteration is maternally inherited, via Sanger sequencing (Fig. 1A, B). The pG608R affects an evolutionarily conserved residue located within the C-terminal regulatory domain of the *ELF4* protein and might interfere with local conformation and stability (Fig. 1C, D). To further delineate the structural consequences of the mutation, we performed a comparative analysis of the wild-type Gly608 and mutant Arg608 residues using the AlphaFold-predicted ELF4 structure (UniProt Q99607). Glycine, lacking a side chain, confers conformational flexibility within protein domains. In contrast, arginine introduces a bulky, positively charged guanidinium-containing side chain. This substitution is predicted to alter local steric configuration, increase hydrogen bond donor capacity, and modify electrostatic surface potential. Structural modeling suggests that the introduction of Arg608 may disrupt local packing density and generate novel electrostatic interactions that are absent in the wild-type configuration (Fig. 1D). Such physicochemical alterations are known to influence protein stability and interfaces involved in intermolecular interactions [9]. Furthermore, in silico prediction tools, including CADD, SIFT, PolyPhen-2, and Mutation Taster, classify the variant as deleterious; it has not been previously found to be associated with any disease or listed in public genome databases (Table S1).

## Results

Flow cytometry of PBMCs from the patient and healthy control (HC) demonstrated an expanded CD8 + T cell population and a skewed CD4/CD8 ratio (Fig. 1E). Both CD4 + and CD8 + T cells in the patient’s PBMCs were elevated compared to those of an age- and gender-matched healthy individual (Fig. 1E and S1). CD4 + subsets displayed diminished CD45RA, CD25 and CD44 expression in the patient, while CD62L expression showed no significant change (Fig. 1F and S2). Furthermore, we sought to assess T cell differentiation and activation status by examining the expression of CD45RA, CD44, CD25, and CD62L on CD4^+^ and CD8^+^ T cells in peripheral blood mononuclear cells (PBMCs) from the patient (PT) and the healthy control (HC), under both basal and anti-CD3/CD28-stimulated conditions (Fig. 1G and S3–S5). In the unstimulated state, CD4^+^ T cells from both the patient and healthy control (HC) were primarily CD45RA^+^ and CD44^low,^ indicating a largely naïve CD4^+^ pool. After αCD3/αCD28 activation, the proportion of CD45RA^+^ cells declined, with levels persisting markedly lower in the patient. Concordantly, expression of CD44 increased prominently in both the patient and healthy control, along with greater elevation in the patient (Fig. 1G and S4). In parallel, the frequency of CD44^+^CD62L^+^ central-memory-like cells increased in both HC and the PT compared to the basal situation, with a greater increment in the patient. This shift was accompanied by a decline in CD45RA^+^ (Fig. 1G and S4) cells and a corresponding rise in the CD45RA^⁻^ compartment (Figure S4). Further, upon stimulation, expression of CD25 was prominently upregulated, confirming robust activation (Fig. 1G). Both the patient and healthy control (HC) demonstrated low CD25 and CD44 expression within CD8^+^ T cells under basal conditions, with a predominance of CD45RA^+^ cells (Fig. 1G and S5). Upon αCD3/αCD28 stimulation, CD8 + T cells from both the patient and healthy control demonstrated up-regulated CD25 expression (Fig. 1G and S5). Concordantly, a significant expansion in CD44^+^ cells and the CD44^+^CD62L^+^ compartment was observed (Fig. 1G and S5). Furthermore, we assessed the NK cell-mediated cytotoxicity in the patient under both unstimulated and IL-15-stimulated conditions. At baseline, we demonstrated that both the frequency and MFI of Granzyme B-producing NK cells were lower in the patient than in the healthy control. Upon stimulation, Granzyme B expression increased in HC, although a significant change was not observed in the PT (Fig. S6). Similarly, the healthy control has demonstrated higher perforin levels than the patient after IL-15 stimulation (Fig. S6). Additionally, sorted NK cells from the patient and healthy control were co-cultured with labeled K562 target cells at distinct effector: target (E:T) ratios of 1:0, 1:1, and 1:2. Then, cytotoxicity was quantified as the percentage of Annexin-V^+^ and/or 7-AAD^+^ cells within the target gate. While NK cells from the healthy control demonstrated prominent, ratio-dependent killing, NK cells of the patient showed lower killing ability against the target (Fig. 1I). Next, labeled PBMCs were cultured for 72 h under the indicated stimuli, and proliferation was quantified as %CFSE-low cells in CD4 + and CD8 + and lymphocyte gates. (Fig. 1H and S8). Higher proliferation was observed in the patient’s CD4^+^ T cells than those from the healthy control following PI and CD3/CD28 stimulation, even though no significant difference was indicated for PHA or IL-2 (Fig. 1H and S8). A similar trend has been noted for the proliferation of CD8^+^ T cells and total lymphocytes (Fig. 1H and S8).

## Discussion

We present a case of an adolescent patient with clinical and cellular immunological findings suggestive of decreased ELF4 activity, resulting in a combined impairment of T- and NK-cell function. Similar to half of the previously reported cases of Deficiency in *ELF4*, X-linked, our patient did not present with recurrent infections (11 out of 23), enteritis, or mucosal inflammatory manifestations (9 out of 23), suggesting phenotypic variability associated with *ELF4* variants [1, 3]. This finding supports the hypothesis that cytotoxic failure and aberrant lymphocyte homeostasis are monogenic outcomes of Deficiency in *ELF4*, X-linked. Mechanistically, phenotype aligns with loss of ELF4’s dual roles: restraining T-cell activation and licensing NK-cell maturation. The structural replacement of a conformationally permissive glycine with a positively charged arginine could provide a mechanistic basis for the observed immune phenotype, as such substitutions can alter protein stability and regulatory interactions. The creation of *ELF4*^−/−^ mice demonstrated substantial developmental and functional impairments in NK cells, and these functional defects have been attributed to the capability of *ELF4* to bind the perforin promoter, which is vital for sustaining perforin’s basal expression, accompanied by a reduced number of NK cells in patients with Deficiency in *ELF4*, X-linked [5]. Further, prior research demonstrated that *ELF4* interferes with the proliferation of naïve and effector CD8^+^ T cells and constrains Th17 differentiation from CD4^+^ naïve T cells within an experimental autoimmune encephalomyelitis model [6, 8]. Consistent with these observations, in our case, while the reduced granzyme/perforin content and impaired killing after IL-15 reflect the hallmark NK-cell maturation defect, the patient’s skewed CD4/CD8 ratio, rapid acquisition of memory-like traits, and hyperproliferation after stimulation are consistent with the release of the ELF4–KLF4 checkpoint. It’s noteworthy to point out that *ELF4* deficiency also weakens interferon amplification downstream of STING/MAVS, which also compromises innate antiviral responses [7]. This suggests that vulnerability to viral pathogens extends beyond lymphocyte dysfunction. However, clinical manifestations are still rather limited despite this extensive mechanistic role, which is consistent with earlier reports that highlight mucosal inflammation and recurrent viral infections [1]. This implies that *ELF4* is redundant for many immune processes but nonredundant for the control of cytotoxic lymphocytes and interferon pathways. Although one blood sampling was done four weeks post-immunomodulatory therapies, including glucocorticoids, intravenous immunoglobulin, and rituximab, the consistency of functional alterations across independent assays at different time points supports that the observed immune phenotype likely reflects intrinsic ELF4-associated immune dysregulation rather than transient treatment-related effects.

A key limitation of this study is the lack of direct molecular functional validation of the *ELF4* variant at the protein or activity level. Although cellular functional assays revealed consistent defects in T- and NK-cell compartments consistent with prior literature, further studies, including protein expression analyses and transcriptional activity assays, are required to definitively establish the pathogenicity of the Gly608Arg variant.

In conclusion, this study expands the phenotypic and functional spectrum associated with *ELF4* variants and suggests that the identified variant may contribute to immune dysregulation characterized by T-cell hyperactivation and impaired NK-cell cytotoxicity.

## Supplementary Information

Below is the link to the electronic supplementary material.

Supplementary Material 1

## Data Availability

No datasets were generated or analysed during the current study.
