# Supplementary material for: A novel ELF4 gene variant disrupts T and NK cell function in a patient with immune thrombocytopenia (ITP)
Source: Inflamm Res. 2026 May 19;75(1):115. doi: 10.1007/s00011-026-02270-1 (PMC13183722; doi:10.1007/s00011-026-02270-1)
Supplement: Supplementary file 1 — Supplementary Material 1 [file 11_2026_2270_MOESM1_ESM.pdf]

**Electronic Supplementary Material**

# A Novel *ELF4* Gene Variant Disrupts T and NK Cell Function in a Patient with Immune Thrombocytopenia (ITP)

Perihan Kader Kendirli[1,2], Şerife Erdem[2,3], Ayşenur Paç Kısaarslan[4], Veysel Gök[5], Eda Kayhan[4], Alper Özcan[5], Muhammet Ensar Dogan[6], Christoph Klein[7,8], Ekrem Ünal[9] and Ahmet Eken[2,10,11,*]

[1]Department of Bioengineering, Institute of Engineering and Natural Sciences, Abdullah Gül University, Kayseri, Türkiye

[2]Genome and Stem Cell Center (GENKOK), Erciyes University, Kayseri, Türkiye

[3]Department of Immunology, Faculty of Medicine, Kırşehir Ahi Evran University, Kırşehir, Türkiye

[4]Division of Pediatric Rheumatology, Faculty of Medicine, Erciyes University, Kayseri, Türkiye

[5]Division of Pediatric Hematology and Oncology, Faculty of Medicine, Erciyes University, Kayseri, Türkiye

[6]Division of Medical Genetics, Kayseri City Hospital, Kayseri, Türkiye

[7]Department of Pediatrics, Dr. von Hauner Children's Hospital, University Hospital, Ludwig Maximilian University of Munich, Munich, Germany

[8]German Center for Child and Adolescent Health (DZKJ), Munich Site, Munich, Germany

[9]School of Health Sciences, Hasan Kalyoncu University, Gaziantep, Türkiye

[10]Department of Medical Biology, Faculty of Medicine, Erciyes University, Kayseri, Türkiye

[11]Department of Immunology and Microbiology, University of Colorado Anschutz Medical Campus, Colorado, USA

*Correspondence:
Ahmet Eken, PhD
E-mail: ahmet.eken@gmail.com

**This electronic supplementary file includes:**

Supplementary Materials and Methods

Supplementary Table 1

Supplementary Figures 1,2,3,4,5,6,7,8

## SUPPLEMENTARY MATERIALS AND METHODS

### Whole-exome sequencing (WES) and Variant Analysis

Genomic DNA (gDNA) was isolated from the whole blood of the patient and his parents and then used for whole-exome sequencing. WES was carried out at the NGS facility of Dr. von Hauner Children's Hospital in Munich, Germany. Exome capturing was conducted utilizing the *SureSelect XT Human All Exon V5+UTR or V6+UTR kits (Agilent Technologies, USA)* to generate indexed libraries. Following that, these libraries were sequenced using *the Illumina NextSeq 500 platform,* yielding an average read depth of roughly 100× throughout the exome. To prioritize rare pathogenic variants, downstream bioinformatic analysis entailed variant calling and filtering. Identified ELF4 mutations were then confirmed via Sanger Sequencing.

### Human Samples and PBMC Isolation

Peripheral blood samples were collected from the patient and a healthy donor, matched by age and gender, and peripheral blood mononuclear cells (PBMCs) were isolated from the whole blood using a density gradient with Lymphoprep (Secoll #RLL-001) according to the manufacturer's instructions. Then, PBMCs were counted using a hemocytometer following viability assessment with Trypan Blue (Cat# SKU: 03-102-1B).

### TCR Activation and Cell Surface Staining

PBMCs resuspended at $2 \times 10^5$ cells/mL with Dulbecco's phosphate-buffered saline (DPBS, with no calcium or magnesium; Gibco) supplemented with *%2 FBS. Cells* were then stained with monoclonal antibodies against surface proteins, including *PE-CD4 (Biolegend, #980804), APC-Cy7-CD8 (Biolegend, #344714), FITC-CD45RA (Biolegend #983002), Pacific Blue-CD25 (Biolegend #302627), PerCp-Cy5.5-CD44 (Biolegend #397520),* and *Brillant Violet 510-CD62L (Biolegend, # 304844).* 20 µL of Fc receptor blocking reagent was added to each sample, followed by incubation on ice for 10 minutes. After five minutes of centrifuging the cells at 1500 rpm, the supernatant was immediately discarded. PE-CD4, APC-Cy7-CD8, FITC-CD45RA, Pacific Blue-CD25, PerCp-Cy5.5-CD44, and Brillant Violet 510-CD62L were subsequently targeted by monoclonal fluorophore-conjugated antibodies. The same surface staining procedure was repeated after 3 days of activation with anti-CD3 mAb (1 µg/ml) and anti-CD28 mAb (1 µg/ml).

### Cytotoxicity Assay

According to the manufacturer's protocol, K562 cells were labeled with Tag-it Violet (BioLegend, #425101). Subsequently, NK cells sorted from the peripheral blood mononuclear cells (PBMCs) of the patient and healthy control by a human NK Cell Isolation Kit (Miltenyi

Biotec, Bergisch Gladbach, Germany) following the manufacturer's instructions were cocultured with the labeled K562 cells at a density of 20,000 cells per well. The co-culture was maintained at 37°C for 4 hours under three distinct K562:PBMC ratios: 1:0, 1:1, and 1:2. Following the manufacturer's instructions, cells were stained with 7-AAD using the FITC Annexin V Apoptosis Detection Kit (BioLegend). A FACSAria III flow cytometer was then utilized to gate the labeled K562 cells and analyze for apoptotic markers.

To evaluate intracellular perforin and granzyme-B levels, NK cells were further purified via negative selection using a human NK Cell Isolation Kit (Miltenyi Biotec, Bergisch Gladbach, Germany) following the manufacturer's instructions. The cells resuspended as 1 × $10^6$ cells/mL in complete RPMI 1640 medium (Thermo Fisher Scientific, USA) supplemented with 10% fetal bovine serum (FBS; Gibco) and 1% penicillin/streptomycin were incubated for 16 hours at 37 °C in 5% $CO_2$ in two distinct culture conditions as unstimulated and stimulated with recombinant human IL-15 (10 ng/mL; PeproTech, USA). Following incubation, cells were washed twice with Dulbecco's phosphate-buffered saline (DPBS, without $Ca^{2+}$/$Mg^{2+}$; Gibco) and stained with monoclonal antibodies anti-CD3 and anti-CD56. Cells were then fixed and permeabilized with the Cytofix/Cytoperm kit (BD Bioscience, USA) followed by intracellular staining with anti-Perforin and anti-Granzyme B monoclonal antibodies (BD Biosciences).

**CFSE Labeling of Lymphocytes**

PBMCs were labelled with 5 μM carboxyfluorescein diacetate succinimidyl ester (CFSE) in pre-warmed phosphate-buffered saline (PBS) containing 5% fetal bovine serum (FBS) for 5 minutes at room temperature. After incubation, cells were washed once with Dulbecco's phosphate-buffered saline (DPBS; calcium and magnesium-free; Gibco). Subsequently, cells were seeded at a density of 2 × $10^5$ cells per well and 4 distinct stimulant conditions were applied to the triplicated cultures: phytohemagglutinin (PHA) (5 μg/mL), anti-CD3 (1 μg/mL) in combination with anti-CD28, IL-2 (2 ug/mL), PI (5 ug/mL). Upon 72 hours of incubation, a FACSAria III flow cytometer was utilized to analyze the cells, and FlowJo software was used to process the data.

**Statistical Analysis**

Experimental results were analyzed and visualized by using GraphPad Prism software (USA). One-way and unpaired Student's *t* test were used to assess statistical significance, where significance levels were denoted by asterisks (* $P < 0.05$, ** $P < 0.01$, *** $P < 0.001$, **** $P < 0.0001$). Non-significant (ns) P values were defined as those over 0.05.

**Table S1.** In silico prediction and population frequency assessment of the ELF4 p.Gly608Arg.

| Gene | Variation | | | In Silico Predictions | | | | GnomAD AF | |
|---|---|---|---|---|---|---|---|---|---|
| | Transcript | HGVS C | HGVS P | CADD | Polyphen2 | SIFT | Mutation Taster | v2.1.1 | v.4.0 |
| *ELF4* | ENST00000308167 | c.1822G>C | p.Gly608Arg | 25.4 | 0.634 | 0.998 | D | - | - |

B: Benign, D: Deleterious.

Regarding CADD v1.7, variants with scores above 20 were classified as damaging.

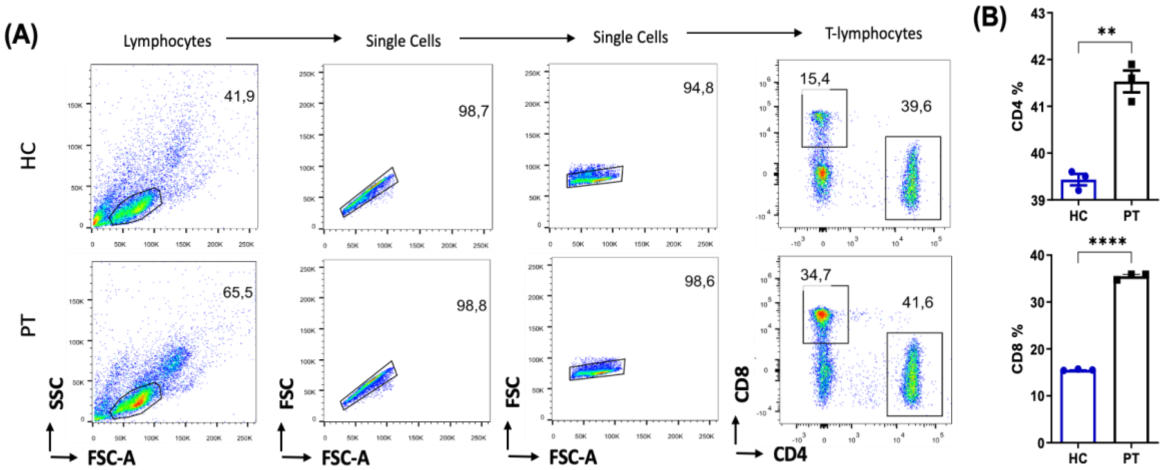


**Figure S1. (A)** Gating strategy for T lymphocytes. **(B)** Frequencies of $CD4^+$ T and $CD8^+$ T cells in the patient compared to gender-matched healthy control (HC).

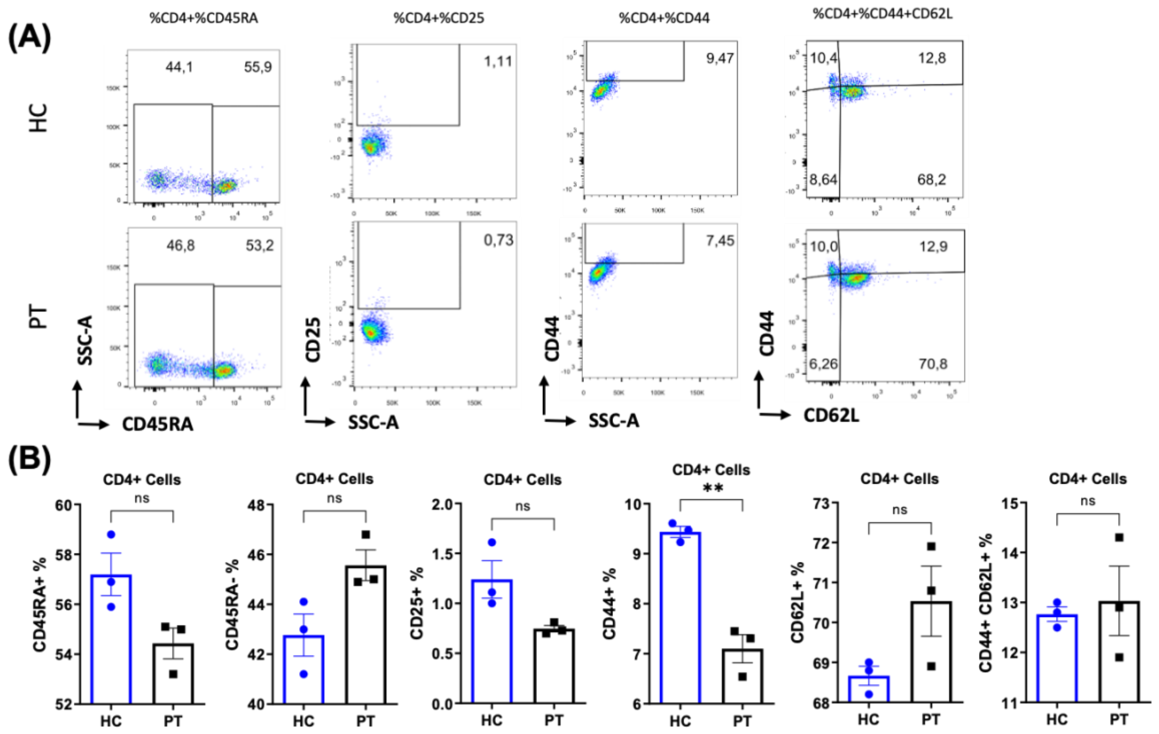


**Figure S2.** Immunophenotyping of CD4$^+$ T cells from the patient (Pt) and healthy control (HC). **(A)** Representative flow cytometry plots demonstrating the expression of CD45RA, CD25, CD44, and CD62L on CD4$^+$ T cells from the patient (Pt) and an age and gender-matched healthy control (HC). **(B)** Percentages of CD4$^+$ T cell subsets for CD45RA$^+$, CD45RA$^-$, CD25$^+$, CD44$^+$, CD62L$^+$, and CD44$^+$CD62L$^+$ comparing PT and HC.

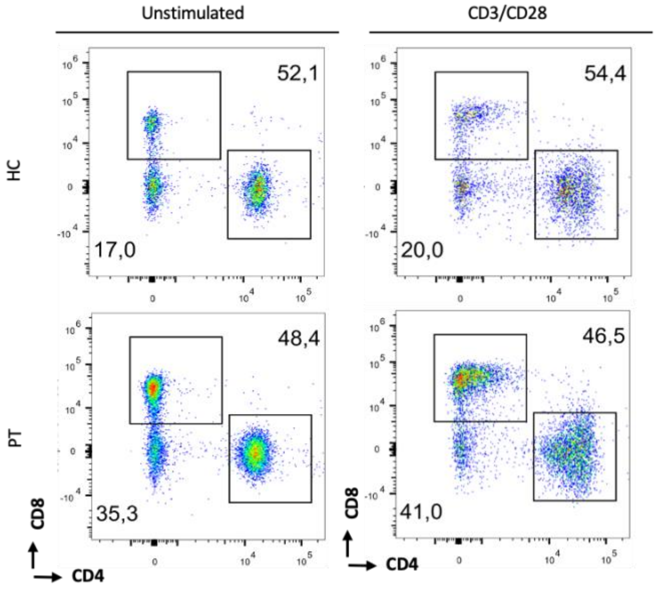


**Figure S3.** Representative flow cytometry plots showing preserved CD3$^+$/CD4$^+$ T cells proportion after CD3/28 activation.

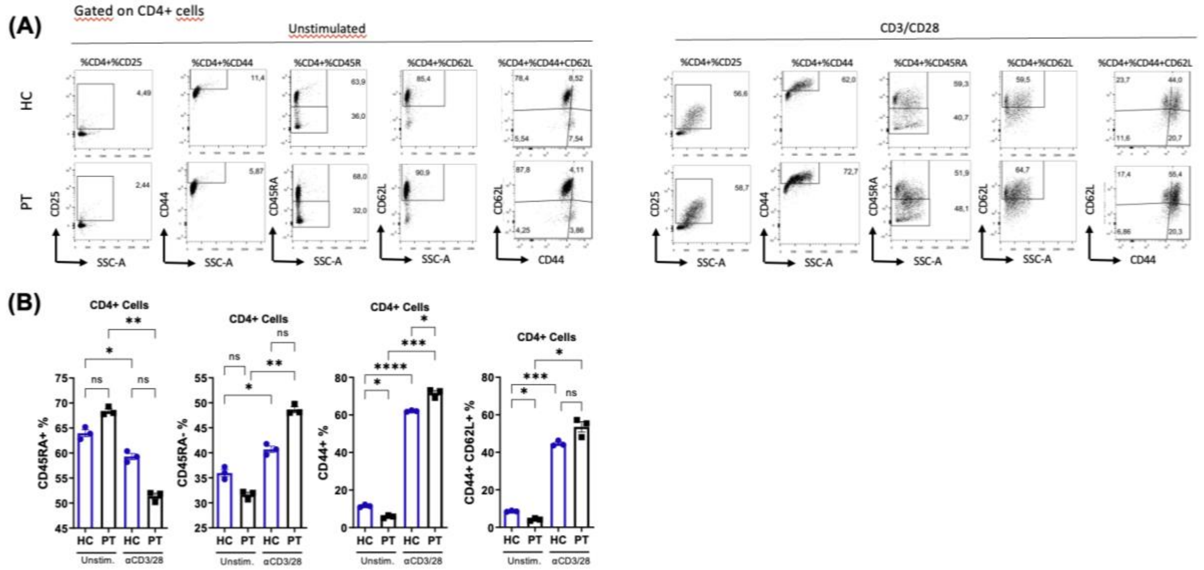


**Figure S4.** Immunophenotyping of CD4⁺ T cells from the patient (Pt) and healthy control (HC) after αCD3/CD28 activation. Representative flow cytometry plots **(A)** and quantified bar graphs **(B)** are shown.

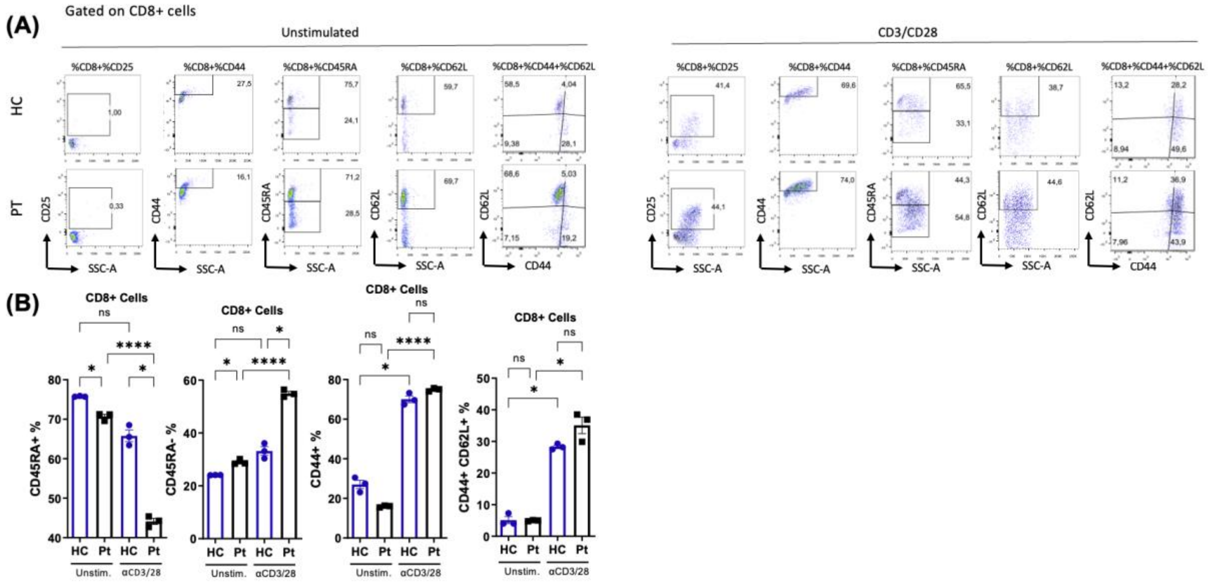


**Figure S5.** Immunophenotyping of CD8⁺ T cells from the patient (Pt) and healthy control (HC) after αCD3/CD28 activation. Representative flow cytometry plots **(A)** and quantified bar graphs **(B)** are shown.

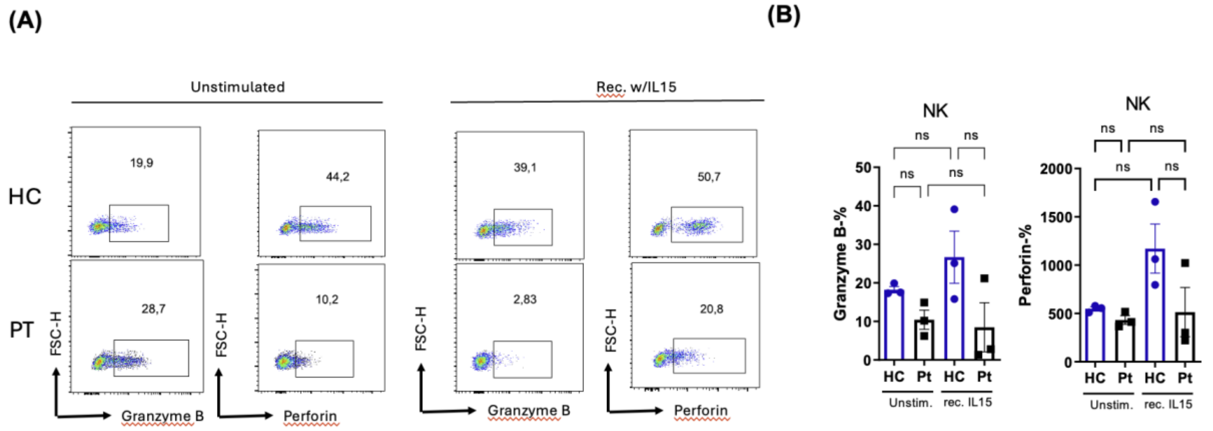


**Figure S6.** Flow cytometry analysis of sorted NK cells from the patient (PT) and a healthy control (HC) under unstimulated and IL-15–stimulated conditions. Representative plots **(A)** and quantified bar graphs **(B)** illustrate the Granzyme B$^{+}$ and Perforin$^{+}$ NK cells from the patient (PT) and a healthy control (HC).

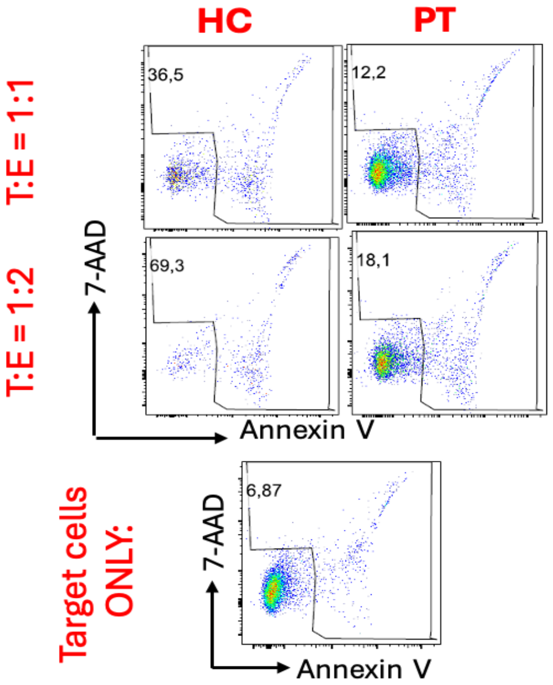


**Figure S7.** Gating strategy for the NK cell cytotoxicity assay, demonstrating the identification of NK cells within PBMCs from the patient (PT) and an age- and sex-matched healthy control (HC).

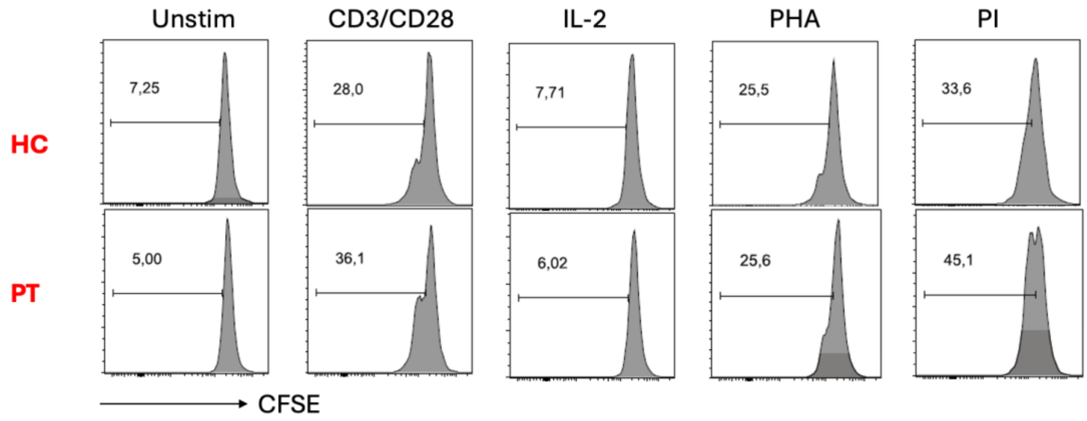


**Figure S8.** Representative CFSE-based proliferation histograms of PBMCs from the patient (PT) and a healthy control (HC) under different stimulation conditions: unstimulated, CD3/CD28, IL-2, PHA, and PI. Numbers indicate the percentage of proliferating cells.
